# The efficacy of Chinese herbal ointment in treating perianal eczema: A systematic review and meta-analysis

**DOI:** 10.1097/MD.0000000000034397

**Published:** 2023-07-21

**Authors:** Hui Zhang, Minghao Lin, Yiwen Zhang, Shilin Liu, Andong Li, Guofeng Li

**Affiliations:** a Changchun University of Chinese Medicine, Changchun, Jilin Province, China; b The Affiliated Hospital to Changchun University of Chinese Medicine, Changchun, Jilin Province, China.

**Keywords:** Chinese herbal ointment, meta-analysis, perianal eczema, the effective rate

## Abstract

Traditional Chinese herbal ointment has significant curative effect and few side effects in the treatment of perianal eczema (PE). Currently, there is no systematic evaluation on the treatment of PE with traditional Chinese medicine ointment. The current aim is to systematically evaluate the efficacy of traditional Chinese medicine ointment in the treatment of PE through meta-analysis.

**Methods:**

Randomized controlled trials on the treatment of PE with Chinese herbal plaster were included in the meta-analysis, which was searched in Chinese and English databases up to March 1, 2023. The search will be conducted in accordance with the object of PICOS framework. Two research will independently use EndnoteX9 to extract the data and evaluate the quality assessment of included trails. Meta-analysis was performed using Revman5.4.1 provided by Cochrane Collaboration; when the outcome indicator is a dichotomous variable, relative risk (RR) was used as the effect size; when the outcome indicator is a continuous variable, weighted mean difference (MD) was used as the effect size, each effect size should be expressed as 95% confidence interval (CI).

**Results:**

The results of meta-analysis showed that: The total effective rate of PE (RR: 1.22, 95% CI: 1.15, 1.30, *P* < .01; *I*^2^ = 32%, Q = 0.17). The cure rate of PE (RR: 3.37, 95% CI: 2.30, 4.94, *P* < .01; *I*^2^ = 21% Q = 0.26). The recurrence rate of PE (RR: 0.25, 95% CI: 0.13, 0.48, *P* < .01; *I*^2^ = 31%Q = 0.23). Itchy points (MD: 0.04, 95% CI: −0.19, 0.27; *I*^2^ = 26%) Skin damage area (MD: −0.37, 95% CI: −0.56, −0.19; *I*^2^ = 26%). Skin damage form (MD: −0.59, 95% CI: −0.81. −0.36; *I*^2^ = 0%).

**Conclusion:**

A total of 11 articles were included in this study for meta-analysis, and the results showed that Chinese medicine ointment is more helpful in improving the skin lesion area and skin damage form, significantly improve the response rate and cure rate, reduce the recurrence rate. Chinese herbal ointment has guiding significance for clinical practice which deserve to use ointments by further experimental and clinical investigation.

## 1. Introduction

Perianal eczema (PE) is an inflammatory skin disease with a high incidence in industrialized countries.^[1]^ As a branch of eczema, clinical manifestations are diverse. According to incomplete statistics, eczema accounts for 2% to 10% of adults and 20% of children in the world. Eczema can be divided into atopic eczema, allergic eczema and irritant eczema.^[2]^ It is generally believed that eczema can be divided into primary eczema and secondary eczema. The former is caused by complex causes, while the latter is mainly caused by other perianal diseases and stimulated by secretions to form PE. At present, modern medicine has not fully clarified the pathogenesis of PE which has no specific drug treatment and lack of unified treatment standard system. PE as a noninfectious skin disease, allergic skin disease which could spread to perianal skin, perineum, scrotum and other parts.^[3]^ This disease has exudate, recurrent attacks, symmetrical skin disorders, polymorphic damage, the main form of papule, erythema, blister, and poor prognosis which is easy to develop chronic eczema in clinic. It has a serious affect in patients’ quality of life, sleep quality, social interaction, etc.^[4]^ PE has 3 types which can be divided into acute, subacute and chronic types.^[5]^ Conventional treatment of PE mainly uses drugs, physical combination and surgery. Among them, drugs are widely used and are mainly treated with glucocorticoids, antihistamines and immune preparations. Glucocorticoids are still the main local treatment measures for PE.^[6]^ Side effects are grievous; long-term use is easy to cause skin atrophy, and pigmentation, easy to relapse. Traditional Chinese medicine believes that PE belongs to “anal dampness,” “hydrangea style,” “soaking sores,” and “healing prickly heat.”^[7]^ Traditional Chinese medicine treatment of PE includes oral Chinese medicine, fumigation, wet compress, external plaster, etc. In recent years, there have been many clinical studies on the treatment of PE with traditional Chinese medicine poultices, but there is no analysis of the clinical effect of traditional Chinese medicine poultices on PE. The objective of this study was to evaluate the efficacy of Chinese herbal plaster in treating PE and to provide real data for clinical application.

## 2. Materials and methods

The meta-analysis was conducted in accordance with the Preferred Reporting Items for Systematic Reviews and Meta-Analysis (PRISMA) statement. This article is registered with PROSPERO (Registration number: CRD42023405256). These studies do not involve humans or animals for ethical or ethical review. This means that they do not involve any behavior that may cause harm to life, physical or mental health. Therefore, there is no need for patient and public participation, informed consent, or ethical approval in the design, process, and outcome of the study.

### 2.1. Data sources and search strategies

The search will be conducted according to the object of study, participants, interventions, comparison, outcome indicator, type of study framework. A literature search was performed in PubMed, Embase, Cochrane Library, Web of Science, China National Knowledge Infrastructure database (CNKI), VIP, and Wanfang database up to March 2023. The PunMed search strategy was as follows: (((“Eczema”[Mesh]) OR (((((Eczemas [Title/Abstract]) OR (Dermatitis, Eczematous [Title/Abstract])) OR (Dermatitides, Eczematous [Title/Abstract])) OR (Eczematous Dermatitides [Title/Abstract])) OR (Eczematous Dermatitis [Title/Abstract]))) AND ((“Ointments”[Mesh]) OR (((((((((Ointment [Title/Abstract]) OR (Unguents [Title/Abstract])) OR (Salve [Title/Abstract])) OR (Salves [Title/Abstract])) OR (Unguent [Title/Abstract])) OR (Pastes [Title/Abstract])) OR (Paste [Title/Abstract])) OR (Skin Ointment [Title/Abstract])) OR (Ointment, Skin [Title/Abstract])))) AND (randomized controlled trial [Publication Type] OR randomized [Title/Abstract] OR placebo [Title/Abstract]) The search was limited to human studies. No restrictions were applied to language. The reference lists of the retrieved articles were reviewed manually for potential eligible research.

### 2.2. Study selection and data extraction

Studies meeting the following criteria were included: Patients were diagnosed as PE; Randomized controlled trial; Experimental group: Chinese patent medicine ointment, mainly for local topical use. Traditional Chinese medicine ingredients should contain Phellodendron amurense, Scutellaria baicalensis, or borneol and other Qingre Huashi medicine. control group: Routine treatment of western medicine ointment. There was no limit to the duration, frequency, or dose of drug treatment; Human subjects were enrolled regardless of age, nationality, region, or race. The combined underlying disease is not limited.

Studies with the following criteria were excluded: Review and case report; Traditional Chinese medicine ingredients should contain Phellodendron amurense, Scutellaria baicalensis, or borneol and other Qingre Huashi medicine. The control group did not conform to the conventional treatment of western ointment; Duplicated studies. According to the efficacy judgment criteria in the Industry Standard of Traditional Chinese Medicine of China-The Efficacy Standard of the State Administration of Traditional Chinese Medicine, that is, the reduction of total symptom points is divided into 4 grades: effective (curative, effective, effective and invalid). Efficacy criteria: efficacy index (nimodipine method) = (pretreatment score-posttreatment score)/pretreatment score 100%; recovery: 90% efficacy index, skin lesions resolved; efficacy: 60% to 89%; most of the skin lesions subsided, the symptoms significantly, pruritus; efficacy: 20% to 59%; the skin lesions partially subsided, symptoms improved, pruritus were reduced; ineffective: efficacy index < 20%; skin lesions subsided not obvious, and pruritus did not change or intensify. The primary outcome was the total effective rate, cure rate, and recurrence rate. Secondary outcomes include pruritus integral, skin lesion area, and skin lesion morphology. Two authors (Hui Zhang and Minghao Lin) independently extracted data, including characteristics of participants, interventions, and outcome measures, in included studies. Data were inputted using Microsoft Excel and validated by double entry. Disagreements were resolved by discussion.

### 2.3. Quality assessment

Assessment of the methodological quality of each study was conducted by 2 authors independently. The risk of bias as to random sequence generation, allocation concealment, blinding of participants and personnel, blinding of the outcome assessment, analysis of incomplete outcome data, selective reporting, and other biases was assessed by the COCHRANE Collaboration Risk of Bias Tool. REVWAN 5.4.1 software (The Nordic COCHRANE Centre, Copenhagen, Denmark) was used for this assessment.

### 2.4. Meta-analysis

Two researchers used EndnoteX9 to extract data and independently assess the quality of the included trial. Meta-analysis was performed using Revman5.4 (https://training.cochrane.org/online-learning/core-software/revman) provided by COCHRANE Collaboration; when the outcome indicator is a dichotomous variable, relative risk (RR) was used as the effect size; when the outcome indicator is a continuous variable, weighted mean difference (MD) was used as the effect size, each effect size should be expressed as confidence interval (CI). *I*^2^ and *Q* tests, and when *I*^2^ was < 50% and *P* > .1, a fixed effect model was selected considering less heterogeneity in included studies; If *I*^2^ was > 50% and *P* < .1, considering the large heterogeneity of the included studies, the random effect model was used. Conduct sensitivity analysis or subgroup analysis as necessary to explore the source of heterogeneity if necessary.

## 3. Results

### 3.1. Study selection

**Figure 1.**
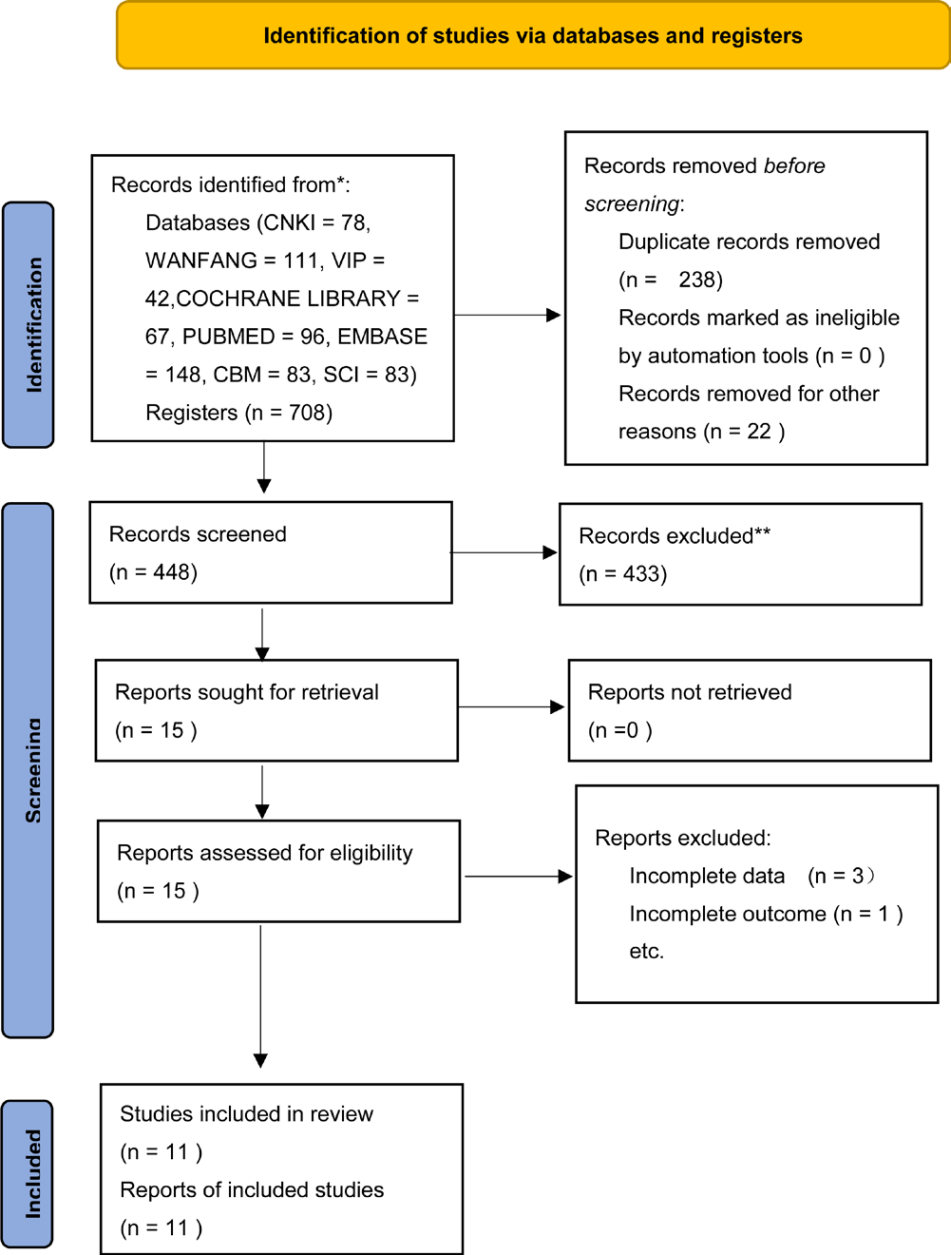
Guidelines flow diagram.

A total of 708 articles were retrieved, CNKI = 78; WANFANG = 111; VIP = 42; CBM = 83; PUBMED = 96; SCI = 83; EMBASE = 148; COCHRANE Library = 67. A total of 314 Chinese articles and 394 English articles were retrieved, and 238 duplicate articles were excluded; Excluding the review, systematic evaluation, and animal experiments 22; By reading the abstract, the study content did not coincide, or the interventions were inconsistent in 433 articles; Excluding 3 articles with lax experimental design; 1 article with inconsistent outcome indicators; Finally, eleven studies were included in the analysis, a total of 808 patients.^[8–18]^ Description of included studies are as follows. (Table 1) We summarized the screening process in our “Study selection flow diagram” (Fig. 1).

**Table 1:** Description of included studies.

Document source	Sample size	man/woman/Number		Mean age/age		Mean course of disease/yr		Intervention	
	T/C	T	C	T	C	T	C	T	C
Zhigang Zhang 2017	30/30	17/13	19/11	40 ± 11.95	39 ± 12.32	-	-	GuiJie ointment	Compound dexamethasone acrtate cream
Bo Zhang 2018	35/35	19/16	21/14	44.20 ± 9.50	43.10 ± 10.40	3.25 ± 1.06	3.12 ± 1.08	Tibetan medicine 25 flavor tea ointment	Desonide cream
Minghong Yu 2013	66/66	-	-	40.5	40.5	1–15	1–15	MUSK Hemorrhoids ointments	Triamcinolone acetonide urea cream
Yongqiang Wang 2014	30/29	14/16	15/14	31.7 ± 3.5	32.6 ± 3.2	0.25 ± 2	0.21 ± 2.5	Moist exposed burn ointment	Triamcinolone acetonide and rconazole cream
Yonghong Shen 2015	30/30	12/18	11/19	81.47 ± 10.22	86.10 ± 8.75	-	-	Huazhi ointment	Calamune Lotion and Compound dexamethasone acrtate cream
Chunsheng Luo 2012	33/30	11/22	10/20	20–85	24–78	10–40	8–35	Longzhu ointment	Compound Beclomethasone Camphor Cream
Zhengzheng Liu 2013	24/24	-	-	73	73	-	-	Baicalein oil cream	Compound dexamethasone acrtate cream
Hongye L *I*^2^ 022	32/31	26/6	27/4	4.02 ± 0.8	4.22 ± 0.8	0.03	0.03	Fuzhiqing ointment	Desonide cream
Li Jin 2019	35/35	18/17	19/16	38.07 ± 11.194	37.17 ± 11.591	13.67 ± 12.745	13.97 ± 11.236	Eczema ointment	Triamcinolone acetonide and rconazole cream
Junhua Jiang 2011	32/31	18/14	16/15	32.48	32.48	0.2 ± 3	0.2 ± 3.2	MEBO moist exposed burn cream	Triamcinolone acetonide and rconazole cream
Rong Ding 2014	60/60	31/29	38/22	42.3 ± 3.5	45.1 ± 4.2	2.6 ± 1.2	2.9 ± 1.3	ChuShi Zhiyang ointment	Desonide cream

### 3.2. Description of included studies

The eleven randomized controlled clinical trials evaluated add-on effect of herbal ointment for PE, most were single center studies. Sample size varied from 48 to 132. The ages of participants were from 3 years to 98 years old. The durations of treatment ranged from 7 days to 3 week. In addition, the basic treatment control groups are as follows: Compound dexamethasone acrtate cream (2) Calamune Lotion and Compound dexamethasone acrtate cream (1). Desnoide cream (3),Triamcinolone acetonide and rconazole cream (3), Triamcinolone acetonide urea cream (1),Compound Beclomethasone Camphor Cream (1).experimental group: GuiJie ointment, Tibetan medicine 25 flavor tea ointment, MUSK Hemorrhoids ointments, Moist exposed burn ointment, Huazhi ointment, Longzhu ointment, baicalein oil cream, Fuzhiqing ointment, eczema ointment, MEBO moist exposed burn cream, ChuShi Zhiyang ointment.

### 3.3. Quality assessment of studies

**Figure 2.**
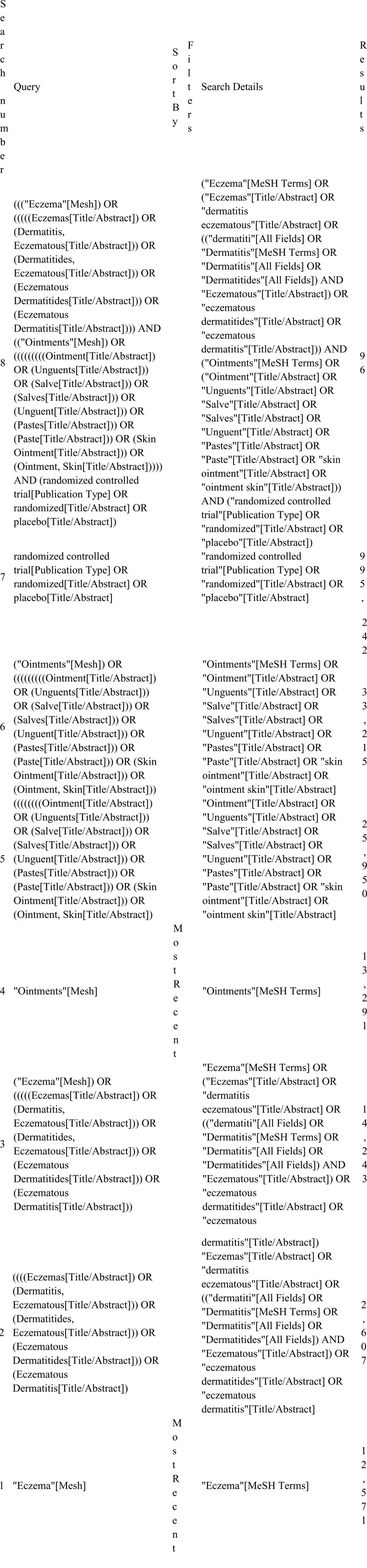
PUBMED search strategy.

**Figure 3.**
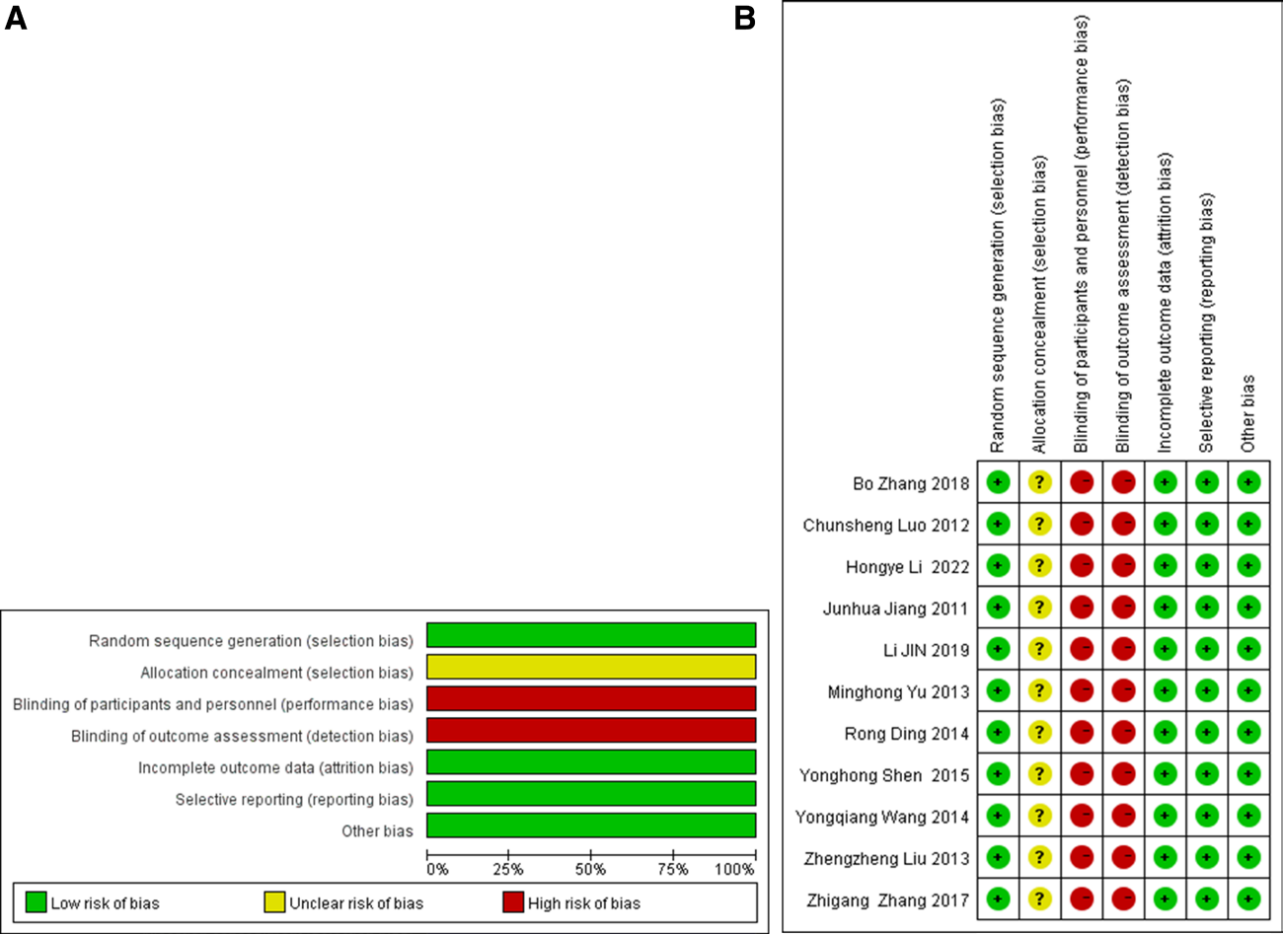
Risk of bias assessment of included studies.

There are 11 articles that reach the risk of a low degree of bias and have high-quality. In the figure, the standard is “+” and not up to the standard for “−”.It is the statistical graph of each item. Based on different drugs and different prices, blindness is less likely to do it, so they are all red. (Fig. 2,3)

### 3.4. Total effective rate of clinical efficacy

**Figure 4.**
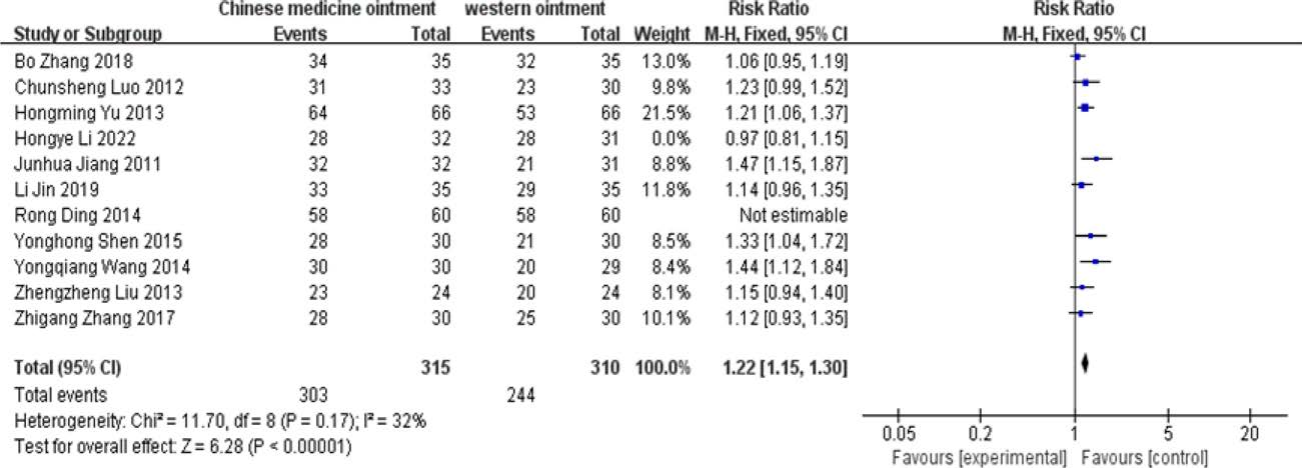
The funnel graph of total effective rate of clinical efficacy.

Heterogeneity test: The overall clinical efficacy evaluation of 11 articles.^[8–18]^ Meta-analysis of effect sizes in 11 publications. For the heterogeneity test, *I*^2^ = 78% > 50%, and *P* < .01 for the *Q* test. Suggesting that the heterogeneity between the literature selected for this study is statistically significant and requires a heterogeneous search. After the sensitivity analysis of the 11 articles, we found that Rong Ding2014 and Hongye L *I*^2^ 022 had a great impact on the heterogeneity. The study was removed and again tested for heterogeneity, showing the remaining 9 publications.^[8,9,12–18]^ Heterogeneity (*I*^2^ = 32% < 50%, *P* = .17 > 0.1), Meta-analysis was performed using a fixed effects model. Summary of 9 studies (RR: 1.22,95% CI: 1.15,1.30, *P* < .01; *I*^2^ = 32%, Q = 0.17). It suggests that the efficacy of Chinese medicine ointment is significantly better than that of conventional treatment in Western medicine. See the forest chart below for details. (Fig. 4)

### 3.5. cure rate

**Figure 5.**
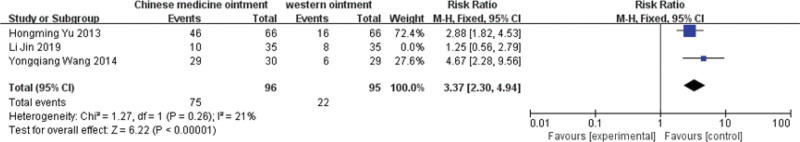
The funnel graph of cure rate.

After 3 studies were included 16, 17, 18, *I*^2^ = 66% and *P* = .05 < 0.1. After sensitivity analysis, Li Jin 2019 had a great influence on heterogeneity. After elimination, *I*^2^ = 21% and *P* = .26, heterogeneity was significantly reduced by the fixed effect model. Three studies had RR values of 3.37,95% CI (2.30, 4.94) and statistically significant Z = 6.22 (*P* < .00001), indicating a higher cure rate for PE. (Fig. 5)

### 3.6. Recurernce rate

**Figure 6.**
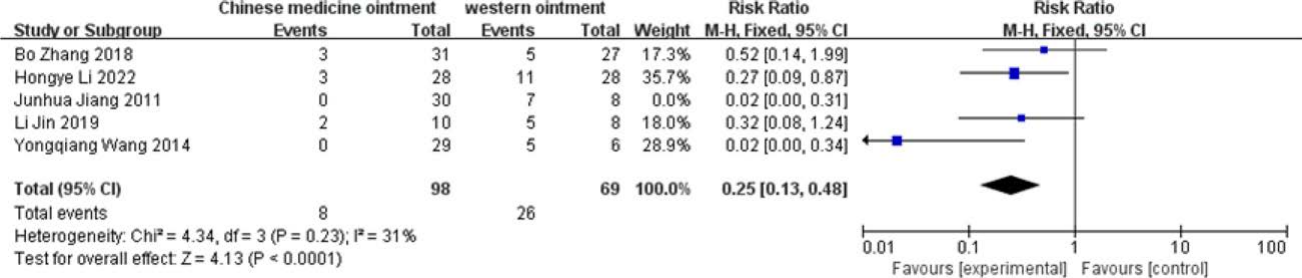
The funnel graph of recurernce rate.

After 5 studies were included,^[8,9,11,17,18]^ *I*^2^ = 51% and *P* = .08 < 0.1 showed significant heterogeneity. After sensitivity analysis, Junhua Jiang had a great influence on heterogeneity. After elimination, *I*^2^ = 31% and *P* = .23, heterogeneity decreased significantly, 5 studies had RR value of 0.25,95% CI (0.13,0.48), and statistical significance Z = 4.13 (*P* < .0001), indicating a lower recurrence rate of PE.(Fig. 6)

### 3.7. Itchy points

**Figure 7.**
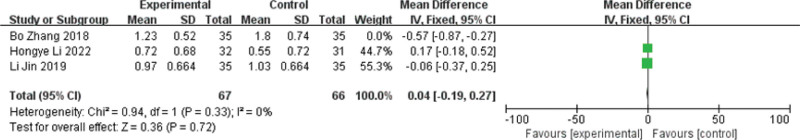
The funnel graph of itchy points.

Three studies included in the analysis,^[9,11,18]^ after heterogeneity test *I*^2^ = 82% and *P* = .004 < 0.1 showed significant heterogeneity., after sensitivity analysis found that Bo Zhang 2018 had a large impact on heterogeneity, *I*^2^ = 0% and *P* = .33, heterogeneity was significantly reduced, fixed effects model for combined analysis, 2 studies summarized MD: 0.04 (95% CI: −0.19,0.27), and no statistical significance (Z = 0.36, *P* = .72). (Fig. 7)

### 3.8. Skin damage area

**Figure 8.**
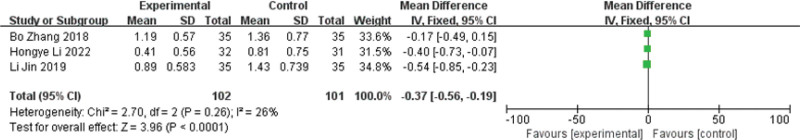
The funnel graph of skin damage area.

Three studies evaluated skin area, tested for heterogeneity,^[9,11,18]^ *I*^2^ = 26% and *P* = .26, no significant heterogeneity, using fixed effect model, 3 studies summarized MD: −0.37 (95% CI: −0.56, −0.19), and statistically significant (Z = 3.96, *P* < .001), suggesting that Chinese ointment can significantly promote wound healing. (Fig. 8)

### 3.9. Skin damage form

**Figure 9.**
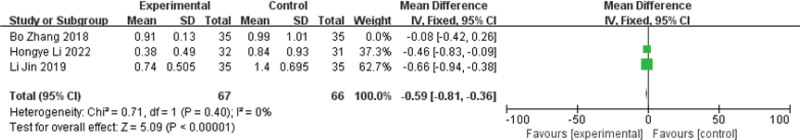
The funnel graph of skin damage form.

A total of 3 studies were included in the analysis;^[9,11,18]^ *I*^2^ = 70%, and *P* = .04 showed significant heterogeneity. After sensitivity analysis, the effect of the heterogeneity *I*^2^ = 0% and *P* = .40, the pooled MD: −0.59 (95% CI: −0.81, −0.81, −0.36), which was statistically significant (Z = 5.09, *P* < .0001), suggesting that Chinese ointment significantly improved skin lesions. (Fig. 9)

### 3.10. The bias test

**Figure 10.**
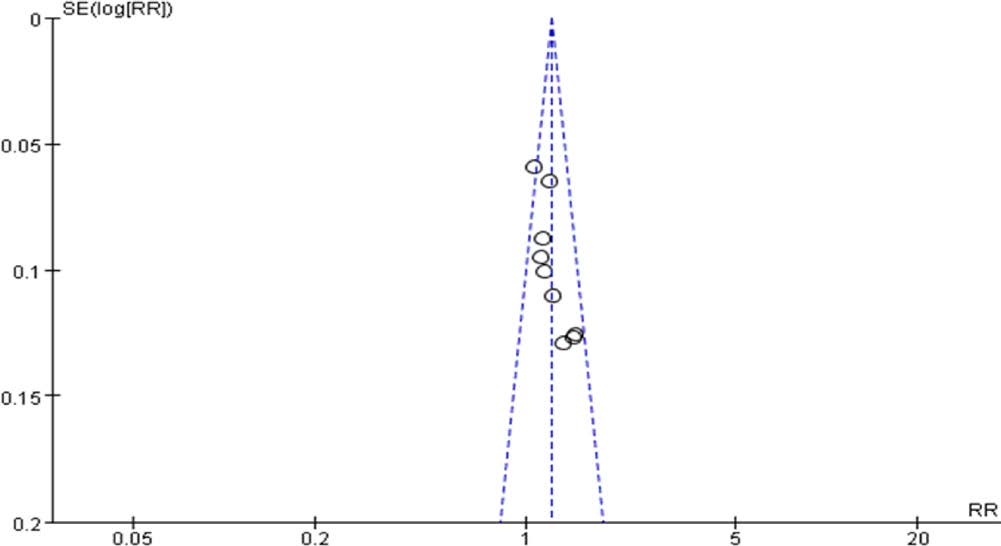
The funnel graph of the bias test.

The study was tested for publication bias by drawing the funnel plot, and the funnel plot symmetry means that there is no obvious publication bias. (Fig. 10)

## 4. Discussion

With the improvement of people’s living standard, the incidence of PE increases year by year. According to modern medicine, PE is an allergic disease, and its pathogenesis has not been fully elucidated. It may be related to metamorphosis, infection, genetics, neurology, anatomy, endocrinology, perianal disease and other factors. The use of glucocorticoids, antihistamines and immunosuppressants is currently the main treatment for PE, but the side effects are more severe and prone to recurrence. Traditional Chinese medicine believes that PE is closely related to “wind, wet and heat.” The Chinese medicine ointment ingredients we choose are Phellodendron amurense, Scutellaria baicalensis, or borneol, which have the common characteristics of clearing heat and dampness. The external use of Chinese ointment can make the drug directly contact with the lesion, and the lesion site can fully absorb the active ingredients of the drug.^[19]^ Direct medicine, direct utility, high compliance, and high safety, which are easy for patients to accept, can be widely used. In recent years, traditional Chinese medicine plaster has been widely used in clinical applications. It has made great progress in promoting transdermal absorption and alleviating local skin irritation. At the same time, it also has advantages of economy, convenience and good curative effect. This study provides theoretical evidence for the advantage of traditional Chinese medicine plaster to improve the clinical effect of PE, and enriching the treatment of PE.

## 5. Conclusion

A total of 11 literatures were included in this study for meta-analysis, and the results showed that in clinical studies, traditional Chinese medicine plaster was more helpful to improve the PE skin area, skin shape and other clinical symptoms, which could significantly improve the effective rate and cure rate, and reduce the recurrence rate. In the included literature, the traditional Chinese medicine plaster containing the effect of clearing heat and drying dampness was taken as a unified whole. Based on clinical studies, the effectiveness of the whole compound was explored. As a promising therapeutic method, Chinese herbal ointment containing heat-clearing and moisture-drying effects can guide doctors to conduct clinical research. However, there are still shortcomings. The intervention measures included in this paper are uneven, and the overall quality evaluation is not high. In the following time, high-quality articles should be selected to better guide clinical practice. In the included randomized controlled studies, a potential bias was identified with regard to blinding and assignment concealment. However, due to the limited trials included trials, this study has some limitations. First of all, the intervention measures of the experimental group are different, and the ointment of the control group is different, which may affect the results of this Meta-analysis. Secondly, the inability to obtain follow-up data in some studies also affects the accuracy of the results. Therefore, more rigorously designed randomized controlled trials are needed in the future to further explore the efficacy and safety of Chinese herbal ointments in the treatment of PE.

Due to the limited experimental data available at present, we are committed to further optimize the experiment in the future, indicating a comprehensive assessment of the quality of the other’s jurisprudence. In order to provide effective evidence for the efficacy of Chinese herbal ointment, further rigorous Randomized controlled trials with long duration of follow-ups are still necessary.

## Author contribution

**Data curation:** Hui Zhang, Minghao Lin.

**Formal analysis:** Hui Zhang, Minghao Lin.

**Investigation:** Yiwen Zhang, Andong Li.

**Methodology:** Guofeng Li.

**Project administration:** Shilin Liu.

**Resources:** Hui Zhang.

**Software:** Hui Zhang.

**Supervision:** Guofeng Li.

**Validation:** Minghao Lin.

**Writing – original draft:** Hui Zhang.

## References

[1] Schauber J Weisenseel P Ruzicka T. Topical treatment of perianal eczema with tacrolimus 0.1%. Br J Dermatol. 2010;161:1384–6. 10.1111/j.1365-2133.2009.09345.x 19575757

[2] Weyandt G Breitkopf C Werner RN. German S1 guidelines for the diagnosis and treatment of perianal dermatitis (anal eczema). J Dtsch Dermatol Ges. 2020;18:648–57. 10.1111/ddg.14125 32469472

[3] Tang Y Sun P Zhang Q. External treatment of perianal eczema [in Chinese]. Med Inf. 2020;33:21–3.

[4] Liu H Xiao J Wu J. Investigation and analysis of the psychological status and quality of life of patients with chronic eczema [in Chinese]. Chin J Dermatol Venereol. 2012;26:332–3.

[5] Expert consensus of TCM diagnosis and treatment of eczema (wet sores) (2016) [in Chinese]. Chin J Dermatol Venereol Integr Tradit Chin West Med. 2018;17:181–3.

[6] Immunology Group of Dermatology and Venereology Branch of Chinese Medical Association. Guidelines for the diagnosis and treatment of eczema (2011) [in Chinese]. Chin J Dermatol. 2011;44:5.

[7] Weng M Fang Z. Clinical observation of 80 cases of damp-humid perianal eczema [in Chinese]. Clin Res Tradit Chin Med. 2020;12:121–3.

[8] Jiang J. Efficacy of MEBO moist burn cream in 32 cases of anal chronic eczema [in Chinese]. Mod Diagn Ther. 2011;22:292–3.

[9] Zhang B Wang S. Clinical observation of 35 cases of chronic eczema with 25-flavor tea gel [in Chinese]. J Gansu Univ Tradit Chin Med. 2018;35:59–61.

[10] Ding R. Treatment of ointment in perianal eczema [in Chinese]. Chin J Lepr. 2014;30:121.

[11] Li H Yang Z Hu X. Observation of topical treatment of perianal eczema after anal fistula in preschool children [in Chinese]. Chin J Anorectal Dis. 2022;42:38–40.

[12] Zhang Z Zou Y Wang Y. Clinical study on the treatment of chronic perianal eczema (blood deficiency type) [in Chinese]. Shizhen Natl Med. 2017;28:1392–4.

[13] Shen Y Lu J Sun G. Observation of clinical nursing efficacy of acute perianal eczema [in Chinese]. Shanghai Nurs. 2015;15:46–7.

[14] Liu Z Wu L. Efficacy of Scutellaria baicalensis ointment on anal eczema in stroke patients [in Chinese]. Inner Mongolia Tradit Chin Med. 2013;32:31–2.

[15] Luo C. Clinical efficacy of dragon Ball ointment applied externally in the treatment of chronic anal eczema [in Chinese]. Chin J Anorectal Dis. 2012;32:64–5.

[16] Yu M. Efficacy observation of Mayinglong Musk hemorrhoid cream in treating perianal eczema [in Chinese]. China Pract Med. 2013;8:159–160.

[17] Wang Y. Analysis of the clinical effect of moist burn cream in 30 cases of anal chronic eczema [in Chinese]. Disabil Med China. 2014:145–145,146.

[18] Jin L Ma B Liang T. Clinical observation of eczema cream for the treatment of perianal eczema [in Chinese]. Pract J Tradit Chin Med. 2019;35:1155–6.

[19] Ping Z Jiang Q Cai J. Overview of perianal eczema [in Chinese]. Pract J Tradit Chin Med. 2021;37:328–30.

